# Applications of optical coherence tomography angiography in glaucoma: current status and future directions

**DOI:** 10.3389/fmed.2024.1428850

**Published:** 2024-09-19

**Authors:** Ruyue Shen, Leo Ka Yu Chan, Amber Cheuk Wing Yip, Poemen P. Chan

**Affiliations:** ^1^Department of Ophthalmology and Visual Sciences, The Chinese University of Hong Kong, Hong Kong, China; ^2^Jet King-Shing Ho Glaucoma Treatment and Research Centre, Department of Ophthalmology and Visual Sciences, The Chinese University of Hong Kong, Hong Kong, China; ^3^Hong Kong Eye Hospital, Hong Kong, China; ^4^Department of Ophthalmology and Visual Sciences, The Prince of Wales Hospital, Hong Kong, China

**Keywords:** optical coherence tomography angiography, glaucoma, vessel density, retinal imaging, glaucoma progression detection

## Abstract

Glaucoma is a leading cause of irreversible blindness worldwide, with its pathophysiology remaining inadequately understood. Among the various proposed theories, the vascular theory, suggesting a crucial role of retinal vasculature deterioration in glaucoma onset and progression, has gained significant attention. Traditional imaging techniques, such as fundus fluorescein angiography, are limited by their invasive nature, time consumption, and qualitative output, which restrict their efficacy in detailed retinal vessel examination. Optical coherence tomography angiography (OCTA) emerges as a revolutionary imaging modality, offering non-invasive, detailed visualization of the retinal and optic nerve head microvasculature, thereby marking a significant advancement in glaucoma diagnostics and management. Since its introduction, OCTA has been extensively utilized for retinal vasculature imaging, underscoring its potential to enhance our understanding of glaucoma’s pathophysiology, improving diagnosis, and monitoring disease progression. This review aims to summarize the current knowledge regarding the role of OCTA in glaucoma, particularly its potential applications in diagnosing, monitoring, and understanding the pathophysiology of the disease. Parameters pertinent to glaucoma will be elucidated to illustrate the utility of OCTA as a tool to guide glaucoma management.

## 1 Introduction

Glaucoma is the most common cause of irreversible blindness worldwide, especially among the elderly population (1). In 2020, glaucoma caused blindness in 3.6 million people aged 50 years and older, accounting for 11% of all global blindness in this age group (2). Primary glaucoma can be broadly divided into primary open-angle glaucoma (POAG) and primary angle-closure glaucoma (PACG) (3). POAG is characterized by an open iridocorneal angle and is often associated with a gradual and chronic increase in intraocular pressure (IOP) due to dysfunction of the trabecular meshwork drainage system, which impedes aqueous humor outflow from the anterior chamber (4). Patients with POAG typically remain asymptomatic until prolonged IOP elevation leads to atrophy of the retinal nerve fibers, resulting in glaucomatous optic disk changes and irreversible visual impairment (5). Normal tension glaucoma (NTG), a subtype of POAG, maintains a consistently low IOP at or below 21 mmHg, which is within the normal range (6). In contrast, PACG is characterized by a narrow or closed iridocorneal angle, often presenting with acute-onset vision loss due to relative pupillary block and anatomical obstruction (5). Although the global prevalence of POAG is six times higher than PACG, both subtypes share similar risk factors, such as advanced age and a positive family history (7). Early diagnosis and prompt treatment are crucial in glaucoma to halt the disease progression and prevent irreversible vision loss. The diagnosis is based on the detection of glaucomatous optic neuropathy and visual field (VF) defects by a comprehensive clinical examination (8). With the advancement of imaging technology, pre-perimetric glaucoma (i.e., glaucoma with detectable glaucomatous optic neuropathy but without visual field defect) became more readily diagnosed. This early detection May improve the management outcome of glaucoma, though it also introduces diagnostic dilemmas.

Optical coherence tomography angiography (OCTA) is a high-resolution imaging technology that allows non-invasive and repeatable examination of the retinal, choroidal, and peripapillary vasculature. OCT produces images by using two beams of coherent light that superimpose to produce an interference pattern based on the difference in their path lengths. The signal is then analyzed to produce the OCT images (9). The principle of OCTA lies in analyzing successive images of the same tissue and detecting the variability of local reflectance signals produced by the motion of red blood cells through the retinal blood vessels (10). In the past, evaluation of the retinal vasculature relied on fundus fluorescein angiography (FFA), which is more invasive, time-consuming (requires time for the dye to reach the retinal vessels for generation of FFA images), and has limited ability to examine the radial peripapillary capillaries (11–13). OCTA has several advantages over FFA, including non-invasiveness, being safe and quick to perform, repeatable, and able to differentiate various layers of retinal capillary plexus. Furthermore, OCTA can provide more diagnostic information with its three-dimensional images and examine the radial peripapillary capillaries that supply the retinal nerve fiber layers (RNFL). With these advantages, OCTA May expand its roles in diagnosis, monitoring, and prognosis in glaucoma management.

The pathogenesis of glaucoma is known to be multi-factorial, with ocular blood flow playing a critical role (14, 15). Glaucoma leads to structural changes in the optic nerve head and retinal nerve fiber layer, which manifest as glaucomatous fundus impairment. As glaucoma progresses, these structural changes are often associated with visual field loss, indicating the severity of the disease. Fundus perfusion refers to the blood flow within the retinal and choroidal vasculature. In glaucoma, reduced perfusion can occur due to various factors, including low diastolic blood pressure and Decreased ocular perfusion pressure (16–22). These changes in blood flow can exacerbate optic nerve damage, leading to further visual field loss (23, 24). Studies have shown a significant correlation between glaucomatous fundus impairment, such as neuroretinal rim thinning and retinal nerve fiber layer loss, and the corresponding alterations in retinal and choroidal perfusion detected by OCTA (25–27). OCTA provides a unique advantage in this context by allowing for the assessment of vascular density and changes in blood flow before visual field defects become apparent (28). The correlation between glaucomatous fundus impairment and changes in fundus perfusion is significant for understanding the disease’s progression. Reduced perfusion can lead to structural changes in the optic nerve, which OCTA can effectively visualize, thereby enhancing the diagnosis and management of glaucoma. This relationship underscores the importance of integrating OCTA into clinical practice for better patient outcomes.

This review aims to provide a comprehensive overview of the application of OCTA in glaucoma. We will explore the role of OCTA in detecting, monitoring, and predicting glaucoma progression, which may enable early intervention for high-risk patients and lead to better clinical outcomes. Additionally, we will discuss the benefits and challenges of applying OCTA and its potential future development, offering insights that May help optimize its utility in glaucoma management and suggest future research directions.

## 2 Methods of literature search

The literature search for this review was conducted in February 2024 using several electronic databases, including PubMed, ScienceDirect, and OpenMD to identify relevant studies in a systematic approach. The search strategy included a combination of keywords including “OCTA,” “glaucoma,” “optic nerve head,” and “vessel density,” and Boolean operators (e.g., “AND,” “OR”) were used to filter appropriate articles. A manual search of the reference lists from pertinent studies was carried out to include any additional papers of interest. The inclusion criteria encompassed English studies examining the application of OCTA in detecting glaucoma severity, disease monitoring, and clinical management. There were no publication year restrictions, and animal studies were excluded. Finally, we included 89 articles in this review.

## 3 Application of OCTA in glaucoma

### 3.1 Optical coherence tomography angiography imaging protocol

OCTA has revolutionized the evaluation and management of glaucoma by enabling detailed visualization of retinal and choroidal microvasculature without the requirement for dye injection. This section outlines the imaging protocols used in OCTA, emphasizing the utility of spectral-domain OCTA (SD-OCTA) and swept-source OCTA (SS-OCTA), the scanning patterns employed, the centering of images, and the most commonly used OCT measurements integrated into OCTA machines for glaucoma assessment.

#### 3.1.1 Utility of SD-OCTA and SS-OCTA

SD-OCTA and SS-OCTA are advanced imaging technologies for visualizing retinal and choroidal vasculature. SD-OCTA utilizes a broadband light source and spectrometer to capture detailed images of the retinal microvasculature with exceptional axial resolution, typically around 5–7 microns. The high-resolution imaging capabilities of SD-OCTA allow for non-invasive visualization of blood flow in the retina and choroid, aiding in diagnosing and monitoring various ophthalmic conditions. However, it is limited by a lower penetration depth and slower scan speeds compared to SS-OCTA (29). SS-OCTA, utilizing a longer wavelength light source and a tunable laser, enables deeper penetration into the choroid and faster image acquisition (30). This technology is particularly beneficial for imaging larger areas with reduced motion artifacts and better penetration of the choroid (31, 32). In studies comparing the two technologies, SS-OCTA consistently yielded larger choroidal neovascularization areas than SD-OCTA, suggesting its superior ability to demarcate the full extent of choroidal vasculature (33).

#### 3.1.2 Centering of OCTA image

**Figure 1:**
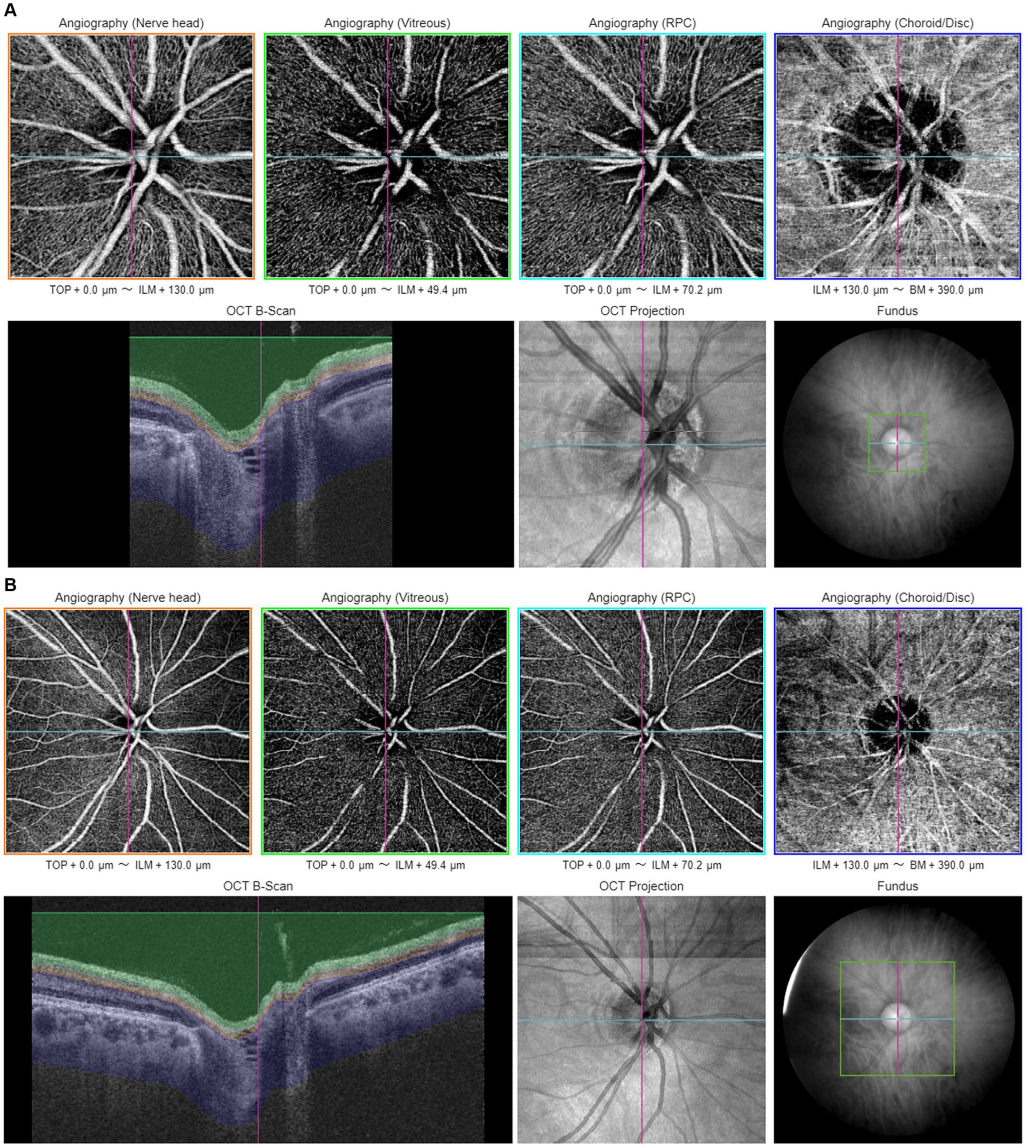
Illustration of optic disc-centered OCTA report captured by Triton DRI-OCT (Topcon, Inc., Tokyo, Japan) with 3 × 3 mm **(A)** and 6 × 6 mm **(B)** scan pattern. ILM, internal limiting membrane; BM, Bruch membrane; RPC, radial peripapillary capillary.

**Figure 2:**
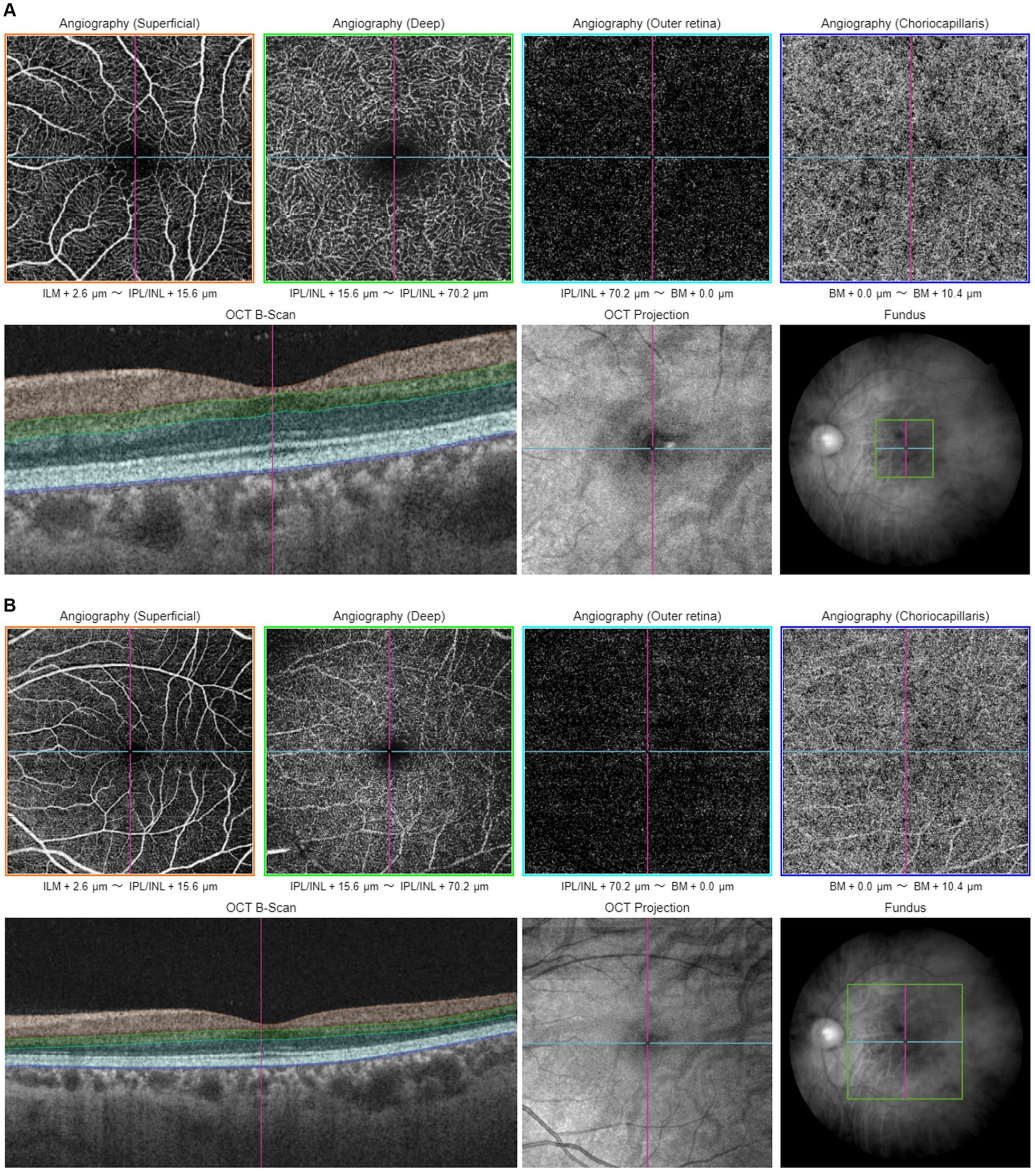
Illustration of macular-centered OCTA report captured by Triton, DRI-OCT (Topcon, Inc., Tokyo, Japan) with 3 × 3 mm **(A)** and 6 × 6 mm **(B)** scan pattern. ILM, internal limiting membrane; IPL, inner plexiform layer; INL, inner nuclear layer; BM, Bruch membrane.

Proper centering of OCTA images is essential for accurate assessment. The optic disk and fovea are the two main areas for OCTA scans in glaucoma. Optic disc-centered scans are centered on the optic nerve head (ONH), providing detailed visualization of the peripapillary microvasculature and RNFL (Figure 1). Studies have demonstrated that OCTA can detect reduced peripapillary vascular flow in glaucomatous eyes compared to healthy controls. Previous studies have reported a 25% reduction in disk flow index in glaucoma patients, with 100% sensitivity and specificity (28, 34). Lee et al. (35) found that areas of Decreased retinal microvasculature in OCTA perfectly corresponded to RNFL defects in glaucomatous eyes. Fovea-centered OCTA scans focus on the macular region, capturing the foveal avascular zone (FAZ) and surrounding retinal layers (Figure 2). They are essential for identifying glaucoma-related alterations in the macular structure. Studies have shown that macular vessel density is lower in exfoliation glaucoma compared to primary open-angle glaucoma, despite similar visual field defects (36). Additionally, foveal avascular zone (FAZ) parameters, such as increased perimeter and Decreased circularity index, are associated with glaucoma severity (37), highlighting the diagnostic value of macular OCTA measurements in differentiating glaucoma types.

#### 3.1.3 Scan patterns of OCTA imaging

Various scan patterns are utilized in glaucoma assessment, such as the 3 × 3 mm (Figures 1A, 2A) and 6 × 6 mm (Figures 1B, 2B) scans, which play a crucial role in diagnosing glaucoma. The 6 × 6 mm scan, particularly its outer sector measurements, showed better diagnostic accuracy for mild glaucoma compared to the 3 × 3 mm scan (38, 39). However, both scan sizes performed similarly for moderate to severe glaucoma (38). Projection-resolved OCTA revealed that glaucoma primarily affects the superficial vascular complex in the macula (40). While the 6 × 6 mm scan May offer diagnostic advantages, the 3 × 3 mm scan demonstrated better repeatability for vessel density, perfusion density, and foveal avascular zone metrics (41). These studies highlight the importance of considering scan size and specific OCTA parameters when evaluating glaucoma.

#### 3.1.4 Common OCT measurements in glaucoma

Several OCT measurements are routinely utilized in glaucoma management and are integrated into most OCTA machines, providing a robust framework for comprehensive evaluation and monitoring of the disease. RNFL is fundamental in diagnosing and tracking glaucomatous damage due to its sensitivity to neurodegenerative changes (42). ONH parameters—including the cup-to-disk ratio, rim area, and disk area–are essential for evaluating structural alterations in the optic nerve head associated with glaucoma. Additionally, the ganglion cell complex (GCC) thickness, encompassing the ganglion cell layer (GCL), inner plexiform layer (IPL), and RNFL, which is crucial for early detection as it reflects the integrity of retinal ganglion cells (43). Macular ganglion cell-inner plexiform layer (GCIPL) thickness shows diagnostic accuracy comparable to RNFL and optic nerve head parameters in early glaucoma (44). Macular thickness measurements provide insights into central vision changes, which are significant in the context of glaucoma. Macular thickness analysis complements RNFL measurements, as the macula contains over 50% of retinal ganglion cells (45). The FAZ area measurement reflects microvascular alterations in the central macula, which can be indicative of the extent of glaucomatous damage.

### 3.2 Quantitative OCTA measurements

**Figure 3:**
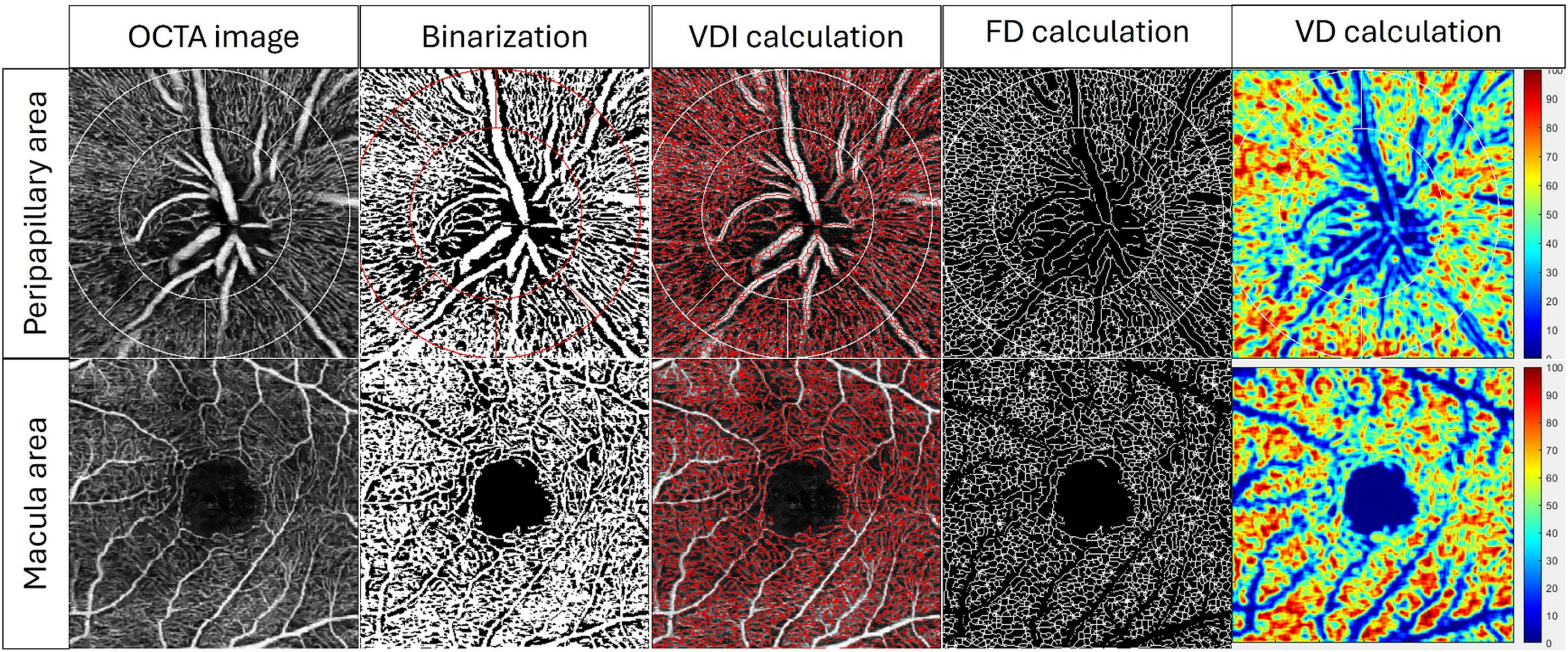
Examples of quantification of OCTA metrics by our customized MATLAB (MathWorks, Natick, MA) program in superficial peripapillary area and superficial macular area. A series of OCTA metrics, including vessel diameter index (VDI), fractal dimension (FD), and vessel density (VD), were automatically calculated from the superficial OCTA image.

OCTA can capture complex retinal capillary images and offers quantitative vascular parameters for both macular and optic disk regions (46). Peripapillary vessel density in the optic region is vital for evaluating glaucomatous damage to the optic nerve and monitoring disease progression (47). On the other hand, macular metrics include measurements such as the FAZ area and macular vessel density, which provide insights into the integrity of the macula and are vital for detecting early glaucomatous changes (48). The built-in capabilities of contemporary OCTA machines can directly measure vessel density and FAZ. However, vessel density alone cannot fully capture all aspects of retinal microvasculature changes, such as vessel branching complexity. To address these limitations, specialized software has been developed to provide a more comprehensive analysis. Our team has developed a customized MATLAB program, which allows us to produce comprehensive OCTA analysis. Figure 3 shows the process of our program to quantify OCTA metrics in both the peripapillary area and the superficial area. A series of OCTA metrics, including vessel density, fractal dimension, and vessel diameter index, were automatically calculated from the superficial slab of the original OCTA image.

#### 3.2.1 Vessel density

Vessel density (VD) is defined as the percentage of area occupied by flowing blood vessels within the selected region of interest (24). In normal eyes, the superficial venous plexus showed a higher VD in the superior (51.4 ± 3.3) and inferior (51.8 ± 3.1) sectors than the nasal (49.9 ± 2.7) and temporal (48.9 ± 3.0) sectors (*p* < 0.001) (49). VD is an implicative parameter in glaucoma eyes. Studies demonstrated that RNFL in normal eyes has a denser microvascular network than glaucoma eyes. Indeed, VD was highest in normal eyes, followed by glaucoma suspects, mild glaucoma, and moderate to severe glaucoma eyes for whole-image VD (wiVD) (wiVD = 55.5, 51.3, 48.3, and 41.7% respectively, *p* < 0.001) and circumpapillary VD (cpVD) (62.8, 61.0, 57.5, 49.6%, respectively, *p* < 0.001) (24).

There was a stronger association between the severity of standard automated perimetry (SAP) mean deviation (MD) vs. cpVD (*R*^2^ = 0.54) and wiVD (*R*^2^ = 0.51), as compared to that between SAP MD vs. RNFL (*R*^2^ = 0.36) and rim area (*R*^2^ = 0.19) (*p* < 0.05 for all) (24). Furthermore, each 1% drop in cpVD was associated with 0.64 dB loss in MD, whereas each 1% Decrease in wiVD was associated with 0.66 dB loss in MD. Even after controlling for the effect of structural loss, there was a significant association between VD and SAP MD.

#### 3.2.2 Fractal dimension

The imagine resolution often limits the measurement of VD. During the VD calculation, a vessel of interest is often missed when the vessel is too small and exceeds the detection limit. In this case, fractal dimension (FD) analysis can be utilized to encounter the problem. To calculate the FD of an image, a count is generated by measuring the number of subunits subtended by a pattern in an image (50). The count is then repeated with smaller subunits. The FD is computed as a ratio that indicates an index of complexity, describing how a particular space of an object can be filled regardless of the influence of scale and resolution limit. Hence, the vascular complexity can be compared between images. Unlike VD, FD is not influenced by the size of the surface plane, allowing a more robust vascularity measurement (50). FD is an invaluable tool for assessing the optic nerve head (ONH) vascularity.

#### 3.2.3 Other OCTA metrics

Vessel area density (VAD) measures the area occupied by vessels divided by the total area, expressed in a percentage (51). VAD provides the best estimation of the real VD because the vessel length and diameter are considered (51). Nevertheless, false negatives on the vascular abnormality can happen when VAD is shown to be unchanged in the context of simultaneous vessel dilation and Decreased perfusion (51). Vessel skeleton density (VSD) only considers whether a vessel exists, regardless of vessel diameter. Because of this, small capillaries and large vessels contribute equally in VSD measurements. VSD is more sensitive than VAD in measuring perfusion changes at capillary levels (51).

Vessel diameter index (VDI) is calculated based on the vessel area map and skeletonized vessel map, by calculating the ratio of VAD to VLD. VDI reflects the average vessel diameter and is sensitive to vascular dilation (51). The foveal avascular zone (FAZ) area is the total number of black pixels enclosed by the FAZ segmentation contour (52). FAZ perimeter length is the length of the perimeter of the FAZ (52). FAZ circularity is the measurement of the degree of similarity of the FAZ to a perfect circle (53). A circularity of 1.0 indicates the shape of FAZ is a perfect circle. The lower the circularity, the less circular the shape of FAZ is.

To enhance the precision in measuring microvascular dropout and identifying potential irregularities within the vascular network, the concept of the intercapillary area (ICA) is employed. The ICA is determined by calculating the average of multiple contiguous regions devoid of vascular structures. This approach allows a more detailed examination of the microvascular landscape, facilitating the detection of areas lacking perfusion that might otherwise be overlooked. By quantifying these non-perfused regions, we can gain more insight into the extent of microvascular compromise, which is critical for understanding the progression of the disease and tailoring appropriate therapeutic interventions (54). The various quantitative metrics of OCTA discussed were summarized in Table 1.

**Table 1:** Definition of quantitative metrics of OCTA.

Parameters	Imaging area	Definition	Built-in system measures
Vessel density (VD)	Optic disk and macula	The percentage of area occupied by blood vessels in a given region (e.g., peripapillary, macular, whole-image) inside the retina or choroid (24).	Yes
Fractal dimension	Optic disk and macula	A measure of the complexity of the retinal or choroidal vasculature. It is calculated by analyzing the branching patterns of the blood vessels, which provides information about the degree of vascular remodeling (110).	No
Vascular tortuosity	Optic disk and macula	A measure of the degree of curvature or bending of blood vessels in the retina or choroid. It provides information about changes in blood flow and vascular resistance in glaucoma (111).	No
FAZ metrics	Macula	The area, perimeter, and circularity of the FAZ could be measured, which are strongly related to the central visual field.	Yes
Area/presence of microvascular dropout	Optic disk and macula	Microvascular dropout refers to the regions with complete loss of choriocapillaris in localized areas of peripapillary atrophy. Its area can be quantified to assess the extent of perfusion defects in the area.	No

### 3.3 Associations between VD and VF measurements

#### 3.3.1 Correlation between peripapillary VD and VF parameters

**Figure 4:**
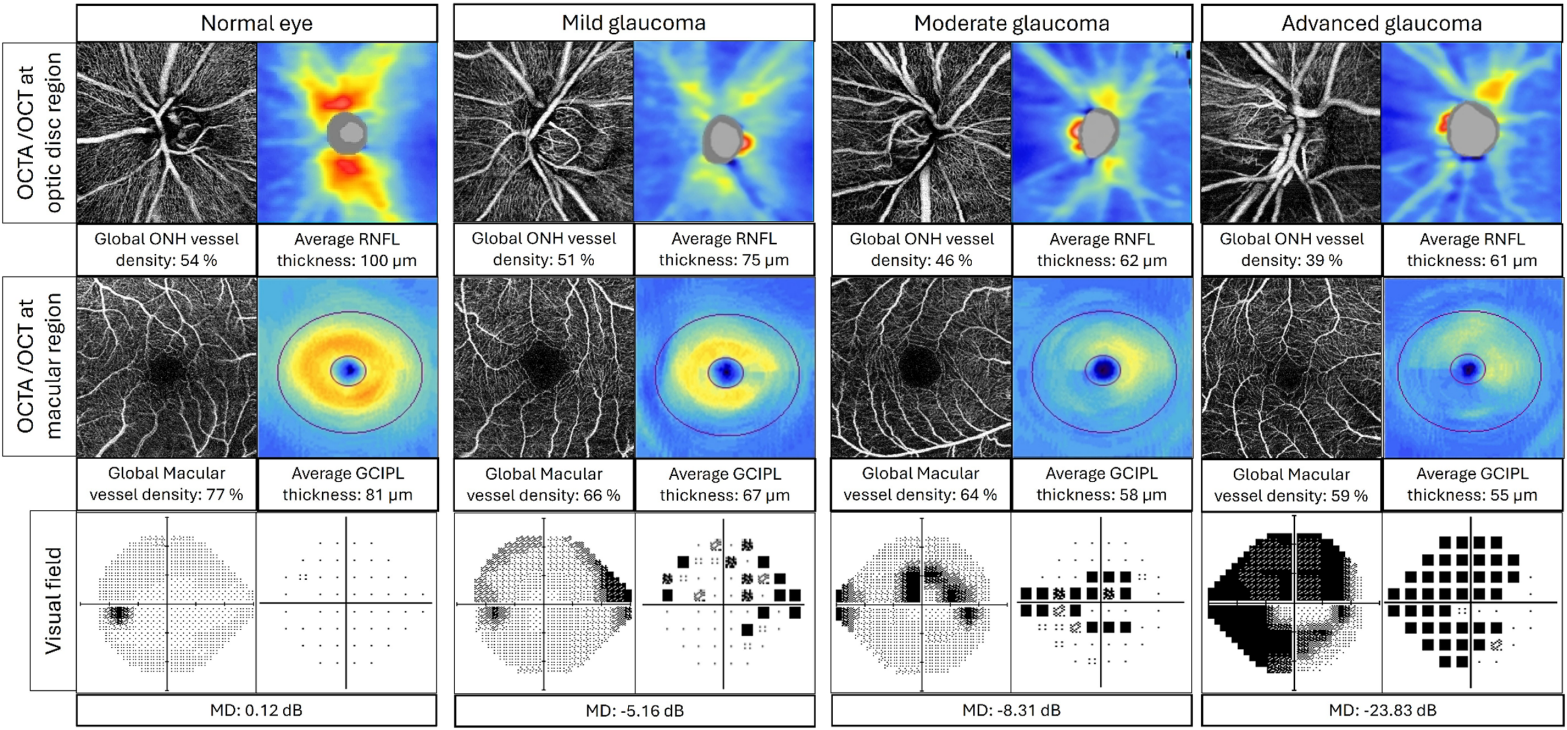
Correlation between OCTA, OCT, and visual field in normal eye, mild glaucoma, moderate glaucoma, and advanced glaucoma. Top layer: OCTA radial peripapillary capillary layer images (left) and OCT RNFL thickness map (right) at optic disk region; middle layer: OCTA superficial images (left) and OCT GCIPL thickness map (right) at macular region; bottom layer: visual field grayscale map (left) and pattern deviation map (right). ONH, optic nerve head; RNFL, retinal nerve fiber layer; GCIPL, ganglion cell inner plexiform layer; MD, mean deviation.

There is a strong correlation between OCTA VD and VF defects in glaucoma (Figure 4). Studies have investigated the relationship between peripapillary VD (pVD) and VF, as well as RNFL thickness. In a study that divided the ONH into 6 sectors, pVD was shown to demonstrate the highest correlation with VF sensitivities (squared semipartial correlation: sr^2^ = 0.17–0.39) (55). Meanwhile, sectoral RNFL thickness showed the highest correlation with VF sensitivity only at the temporal, inferotemporal, and superotemporal sectors (sr^2^ = 0.02–0.34).

A previous study showed that in glaucomatous eyes with single hemifield VF defect, the RNFL and macular ganglion cell complex (GCC) thickness were both diminished even in the intact retinal hemispheres of the perimetrically intact hemifields (56). Hence, pVD can be useful in identifying glaucomatous damage before focal VF defects are detectable. In perimetrically intact hemifields, pVD demonstrated the strongest association with visual field mean sensitivity (*r* = 0.450), followed by perifoveal VD (*r* = 0.403), peripapillary RNFL (*r* = 0.340), and GCC (*r* = 0.290) with *p* < 0.05 for all.

For discriminating pre-perimetric glaucoma and glaucoma suspects from healthy eyes, OCTA detected VD demonstrated comparable discriminant ability with spectral domain OCT (SD-OCT) structural parameters between healthy and pre-perimetric glaucoma eyes (areas under the receiver operating characteristic curve [AUC] were 0.70 vs. 0.66, respectively) (57). Similarly, pVD and RNFL thickness were comparable in discriminating normal and pre-perimetric eyes (AUC 0.880 vs. 0.906, *p* = 0.448) (58). Furthermore, inter-eye VD asymmetry May also be a valuable biomarker to differentiate glaucoma suspects from healthy eyes (2.0% vs. 1.1%, *p* = 0.014) (59).

#### 3.3.2 Correlation between macular VD and VF

Studies have examined the relationship between macular VD and VF sensitivity in glaucomatous eyes. For instance, macular VD was demonstrated to correlate with VF defects, of which the inferior macular VD area and the severity of central VF loss showed the strongest correlation (56, 60). In patients of statistically similar age, glaucoma eyes that exhibited a single-hemifield defect demonstrated a lower macular VD in the intact hemiretina than normal eyes (51.1% vs. 53.8%, *p* < 0.001) (56). The greater the extent of perimetric loss in the hemifields, the higher the degree of structural damage and VD loss in the corresponding hemiretina. The findings reflected that vascular measurements might be a more sensitive indicator than other structural measurements (56). On the other hand, several studies showed that the inner macular thickness had a stronger association with perimetric loss than the inner macular VD (61), suggesting that structural OCT measures May be more correlated with VF loss (61).

### 3.4 Association between VD and OCT measurements

#### 3.4.1 Peripapillary VD and OCT measurements

The next question is whether pVD correlates with other structural OCT measurements and which parameter better discriminate glaucoma eyes from healthy eyes. Indeed, studies have shown a significant correlation between pVD and other structural OCT measurements. A reduced peripapillary microvasculature VD was demonstrated to coincide with the location and extent of RNFL defect observed by red-free fundus photographs in POAG eyes (r = 0.997 and 0.988, respectively, all *p* < 0.001) (35). Another study echoed the findings and showed that wiVD and pVD were highly correlated with the severity of glaucoma, determined by the OCT-measured GCC, RNFL, and rim area (62) – i.e., the more severe the glaucoma, the greater the extent of VD reduction.

The abilities of the OCTA and OCT parameters in discriminating between glaucomatous and normal eyes appear similar. The area under the curves (AUCs) of the OCTA parameters were similar to the corresponding RNFL thickness parameters in the POAG and the PACG groups (*p* > 0.05) (63). In agreement, comparable AUCs of pVD and peripapillary RNFL measurements were also reported elsewhere (64). However, some studies showed conflicting results. For instance, Chung et al. suggested that, while pVD parameters showed similar glaucoma diagnostic ability with circumpapillary RNFL thickness, the inferotemporal and temporal pVDs AUCs were significantly lower than the AUC of RNFL thickness (*p* < 0.05) in early stage glaucoma, suggesting that the VD parameters might have limited clinical value in discriminating early stage glaucoma eye and normal eyes (65). Meanwhile, VF mean deviation (MD) might correlate better with pVD (coefficient of determination, *R*^2^ = 0.54) and wiVD (*R*^2^ = 0.51) than with rim area (*R*^2^ = 0.19) and RNFL thickness (*R*^2^ = 0.36) (all *p* < 0.05) (24).

#### 3.4.2 Macular VD and OCT measurements

The role of macular VD and its correlation with OCT measurements were investigated by many studies. Studies showed a significant correlation between macular VD and macular GCC thickness. Generally, macular VD parameters were reduced in glaucomatous eyes compared with healthy eyes. Chung et al. examined the macular VD in different severity of glaucoma, including early (MD > -6.0 dB), moderate (MD between-6.0 and-12.0 dB), and severe (MD < -12.0 dB) glaucoma. Their results showed that macular VD was significantly reduced in eyes with moderate (VD = 43.78) and severe glaucoma (VD = 40.21), as compared to the control group (VD = 49.83), *p* < 0.001 (65). Other studies showed that macular VD was also reduced in glaucoma suspect, pre-perimetric glaucoma, and early glaucoma eyes (61, 66, 67), with other studies showing mixed evidence (65, 68). Evidence to support the ability of macular VD to discriminate glaucoma eyes from normal eyes is also conflicting. While most studies agreed that macular VD showed a lower AUC than GCC thickness (61, 65, 67), a study demonstrated that superficial macular VD (AUC = 0.757) was better at distinguishing glaucoma suspect eyes (AUC = 0.506) from control eyes (*p* = 0.010) than GCC thickness (66). There was no significant difference between the AUC of macular VD versus OCT GCC in discriminating pre-perimetric glaucoma eyes from control eyes (*p* = 0.856), although OCT GCC was better at distinguishing early glaucoma eyes from healthy eyes (*p* = 0.001) (67).

### 3.5 Differences between NTG, POAG and PACG in OCTA measurements

It was proposed that diminished blood supply to the ONH could be an important pathogenic mechanism of NTG. However, a greater difference in VD was observed between POAG eyes vs. normal controls than that between NTG eyes vs. normal eyes, with a larger AUC value suggesting that the diagnostic ability of pVD was higher in eyes with higher baseline IOP (69). This contradicts what would be expected based on the ocular hypoperfusion-predominant theory – that a greater difference in the VD should have been observed. This might imply that the vascular mechanisms contributing to the pathogenesis of glaucoma are still IOP-dependent; IOP-induced stress and strain seemed to play a central role in the physiology of ONH aging and the pathophysiology of glaucomatous damage (70). Similarly, Scripsema et al. also observed that POAG eyes had a lower pVD than NTG eyes (33.40 ± 6.53% vs. 37.20 ± 3.51%, *p* < 0.05) (71). On the other hand, a study demonstrated that the high-tension glaucoma group exhibited a higher pVD than the NTG group (55.57% vs. 49.78%, *p* < 0.001). These findings indicated a distinct vascular compromise in NTG, even with normal intraocular pressure levels. In PACG eyes, Rao et al. demonstrated significantly reduced retinal VDs in the peripapillary (53.3%, *p* < 0.001) and perifoveal (46.6%, *p* < 0.001) regions (72). Zhu et al. showed that the VD reduction in the peripapillary region was more substantial (11.75%) than that in the perifoveal area (7.55%). They also divided PACG eyes into a well-controlled group (IOP ≤ 21 mmHg) and a non-well-controlled group (IOP > 21 mmHg, despite using maximum tolerated doses of anti-glaucoma medications) (73). It was found that the not-well-controlled group had a more significant reduction of retinal VD and a higher percentage of VD reduction in the peripapillary area (but not in the perifoveal area) than the well-controlled group (74). Table 2 summarizes some studies that document these differences. These differentiations play a significant role in informing clinical assessment and management strategies for these glaucoma types.

**Table 2:** Summary of comparison of OCTA metrics among different subtypes of glaucoma.

		POAG(HTG)	NTG	PACG	Study
Peripapillary/circumpapillary region	VD measurements	Lower global pVD, especially in inferotemporal region		Higher global pVD	Hou et al. (112)
		/	Lower global cpVD	Higher global cpVD	Shen et al. (113)
		Higher mean perfused vessel density (PVD)	lower mean PVDs in all the sectors, except the inferotemporal sector	/	Xu et al. (114)
		Lower pVD	Higher pVD, especially in superonasal region	/	Van Eijgen et al. (115) and Lommatzsch et al. (116)
		Lower average perfused capillary density	Higher average perfused capillary density		Scripsema et al. (71)
	pVD correlation with VF parameters	Has strongest correlation in inferotemporal region		More even correlations across sectors	Hou et al. (112)
		No association	cpVD was significantly associated with the Decrease in VF MD	/	Shen et al. (113)
		Weak correlation	strong correlation between pVD and the contralateral VF sensitivity	/	Zakova et al. (117)
Macular / perifoveal region	VD measurements	Perifoveal VD similar		/	Xu et al. (114)
		Central macular and perifoveal VD did not differ			Hou et al. (112)

OCTA has demonstrated comparable sensitivity and specificity to traditional imaging modalities like fundus photography and standard OCT in detecting glaucomatous changes (63), whereas perifoveal VD exhibits relatively weak diagnostic accuracy (75). However, it May offer additional insights into microvascular alterations that other methods might overlook. For instance, OCTA can visualize the peripapillary and macular microvasculature, providing a more comprehensive understanding of the disease’s impact on ocular perfusion. In PACG, OCTA has shown similar diagnostic capabilities to POAG when accounting for disease severity, reinforcing its utility across different glaucoma types (63). Despite its advantages, OCTA faces limitations specific to each glaucoma type. In NTG, the subtlety of vascular changes can hinder detection, particularly in early stages when RNFL thickness remains normal (54, 76). Furthermore, the reproducibility of OCTA measurements has been questioned, with studies indicating higher coefficients of variation in glaucoma patients compared to healthy individuals, which complicates longitudinal assessments (77). For PACG, the acute nature of the disease can introduce variability in measurements, particularly during episodes of angle closure (78). Additionally, it is essential to consider factors such as patient age, single strength, myopia, and systemic diseases when interpreting OCTA imaging findings (Table 3) (66, 79–84). Future studies exploring the relationship between OCTA results and systemic vascular health could offer insights into the mechanisms underlying glaucomatous damage.

**Table 3:** Factors to consider for analysis of OCTA imaging in glaucoma.

Factors	Effects	Reference
Age of patients	Lower macular and peripapillary VD were observed in older patients.	(66, 79, 80)
Lamina cribrosa (LC) defect	cpVD was reduced to a greater extent in eyes with LC defects compared to those without. There was a spatial correlation between the decreased VD and the location of LC defect.	(81)
Myopia	Peripapillary VD was reduced in myopic eyes.	(82)
Signal strength	VD was reduced in OCTA scans with poorer signal strength.	(83)
Systemic diseases	In patients with hypertension without retinopathy, peripapillary VD Decreased, whereas macular VD increased. In diabetic patients without retinopathy, VD was decreased.	(84)

### 3.6 Monitoring progression of glaucoma

Longitudinal studies were carried out to investigate the role of OCTA in monitoring the progression of glaucoma. In a study that included 100 eyes (32 POAG, 30 glaucoma-suspect, and 38 healthy eyes) followed up for at least 1 year with OCTA, Shoji et al. showed that serial OCTA scans were capable of detecting glaucomatous change in macula VD in eyes (−2.23%/year, *p* = 0.004) without detectable GCC thickness change (−0.44%/year, *p* = 0.609) (79). Compared with healthy eyes, glaucomatous eyes with VF defect demonstrated a greater rate of macular VD loss (−0.76%/year vs. -1.35%/year; *p* = 0.042) and GCC thinning (−0.70 μm/ year *vs*-1.18/ year; *p* = 0.045) (85). Interestingly, macular VD loss was more rapid in POAG eyes vs. pre-perimetric glaucoma eyes (−1.35%/ year vs. -0.93%/ year; *p* = 0.043), whereas GCC thinning rate was similar between the 2 groups (−1.08 μm/year vs. -1.18 μm; *p* = 0.562) (85). Hence, macular VD loss might be a more sensitive biomarker than GCC thinning in this aspect.

Recently, Yoon et al. showed that the reduction rates of the macular VD in the superficial and deep layers were significantly greater in the VF progressors than in the non-progressors (86). In particular, the VD change of the superficial macular layer was related to VF progression or the rate of VF deterioration. This is possible because the superficial layer of macular VD depicts the VD of the superficial vascular plexus that supplies the RNFL and RGCs. In contrast, the deep layer of the macular VD relates to the perfusion of the deep vascular plexus and supplies the horizontal cells in the outer nuclear layer. As the RNFL and RGCs are the primary sites for glaucomatous structural damage, glaucomatous change May be better reflected by the changes in the superficial vascular plexus than in the deep vascular plexus. Therefore, superficial layer macular VD parameters are more predictive of glaucomatous damage and progression. Furthermore, Yoon et al. also demonstrated a significant relationship between serial changes in the superficial layer macular VD loss and concurrent VF progression; there was a linear association between the reduction rates in the superficial layer macular VD parameters and VF progression rate (86).

Previous studies have identified various risk factors for glaucoma progression, such as older age (87), female sex (88), lower diastolic blood pressure (89), long axial length (90), thinner RNFL thickness (91), worse VF mean deviation (87), and higher IOP level (92). For OCTA parameters as a risk factor, Wang et al. showed that eyes with lower superotemporal circumpapillary VD at baseline were associated with a higher risk of glaucoma progression, independent of the above-mentioned risk factors (93). This finding May be relevant, particularly for patients with NTG, where disease progression can occur even with effective IOP management. However, it is important to consider the relationship between OCTA findings and glaucoma natural history in terms of cause and consequence. While OCTA can provide valuable insights into the structural changes associated with glaucoma progression, it is necessary to determine whether these findings are the primary cause of the disease or a consequence of the underlying pathological processes. Further research is needed to establish the temporal relationship between OCTA-detected changes and the development and progression of glaucoma.

## 4 Challenges with OCTA

### 4.1 Image quality issue

It is critical to acknowledge the limitations of OCTA image quality before applying the technology in clinical settings. As mentioned, OCTA visualizes vascular patterns and pathological changes by detecting the motion of red blood cells within vessels. Therefore, any motion artifacts during the imaging process (e.g., patients’ involuntary eye movement) can distort the images, leading to inconsistencies and affecting the precision of interpretation (94). Even slight and inevitable movements, such as breathing, tremors, and micro-saccades, can result in pulsations that give rise to motion artifacts (95). Additionally, projection artifacts occur when superficial structures, such as the blood vessels, are erroneously projected onto the deeper retinal or choroidal layers, producing misleading vascular signals (96). The presence of anterior segment opacities could also lead to shadow artifacts that conceal vascular changes in the retinal layer. Opacities can be attributed to vitreous floaters, cataracts, or insufficiently dilated pupils, which influence signal transmission and subsequently affect the quantification of VD (94). Segmentation artifacts May also occur when there is a misidentification of retinal layers, such as the superficial plexus, the deep plexus, the outer avascular retina, and the choriocapillaris (95).

Apart from the intrinsic limitations related to OCTA technology, patient factors are also conducive to a reduction in image quality. For instance, patients with myopia May have concurrent complications such as retinal pigmentary epithelial atrophy and retinoschisis that interfere with OCTA image formation (94). Moreover, the fibrosis of blood vessels in individuals suffering from fibrotic neovascularization could lead to a slow flow rate of erythrocytes (94). This will prolong the time to acquire multiple OCTA scans, resulting in a higher risk of motion artifacts. For better application of this diagnostic modality in the future, identifying and eliminating the OCTA image artifacts are essential for accurate diagnosis and disease monitoring (95). The development of software and technologies for removing the artifacts are not yet comprehensive and artifacts often remains (94).

### 4.2 Floor effect issue for advanced glaucoma

Detection of glaucoma progression in advanced glaucoma can often be challenging because of the floor effect – difficulties of detecting a further structural change in a thin circumpapillary RNFL by the OCT (97, 98). VD measurements demonstrated lower floors than OCT thickness measurements, particularly in the macular region (99). In other words, glaucoma progression could still be detected by observing a further reduction in VD despite a static OCT thickness measurement in eyes with advanced glaucoma. Indeed, OCTA-measured perifoveal VD was the parameter least likely to reach a measurement floor (99). Hence, in advanced glaucoma, OCTA-measured perifoveal VD May serve as a useful tool for monitoring disease progression. Nonetheless, thickness-based OCT parameters still have advantages in monitoring glaucoma progression as they have more steps to measurement floors, which reflect changes within the dynamic ranges of measurement metrics (98), than OCTA parameters (99). Also, thickness-based OCT imaging is less operator-dependent, labor-intensive, and time-consuming.

### 4.3 Lack of standardized protocol for the use of OCTA in clinical diagnosis and management of glaucoma

One of the stumbling blocks that hinders the full utility of OCTA in the clinical management of glaucoma is the lack of standardization among OCTA instruments, varying protocols for imaging, different techniques of data analyses, and the inconsistent nomenclature, leading to erratic and noncomparable results. Establishing specific acquisition settings, image processing algorithms and interpretation approaches May improve the accuracy and reproducibility of OCTA. It is also important to note that OCTA-measured VD is a surrogate biomarker and not a true retinal blood flow measure. Therefore, it does not directly reflect the retinal blood flow rate. Further efforts are needed to explore more reliable parameters to better reflect the retinal blood flow.

## 5 Future development

### 5.1 Development of handheld OCTA

With the increasing utility of OCTA in glaucoma diagnosis and monitoring, the development of hand-held OCTA has promising potential and various benefits in clinical settings. Recent studies highlighted advancements in OCTA devices, demonstrating features for hand-held operation without compromising imaging quality. These devices incorporate a camera-equipped probe. Real-time OCTA images are displayed on a monitor attached to the probe, allowing more accurate localization of relevant anatomical targets (100). Moreover, these hand-held systems employ innovative techniques, such as fast, automatic focusing with an electrical lens and an extended axial imaging range to better visualize of the deep structures. To further minimize motion artifacts induced by involuntary movements of the operator and the patient in a hand-held system, certain OCTA devices could reduce imaging time to 1–2 s, ensuring reliable imaging results and improving diagnostic sensitivity (100).

The development of hand-held OCTA could broaden the clinical applicability of this advanced imaging technology. Hand-held OCTA provides enhanced accessibility and flexibility, benefiting pediatric patients and individuals with limited mobility who cannot undergo traditional benchtop examinations. A user-friendly handheld OCTA device also requires less technical expertise, making it suitable for emergency rooms and primary care clinics. It also enables point-of-care imaging for patients in remote areas with limited access to ophthalmologic care. These benefits maximize OCTA application in the management of glaucoma, allowing early detection and improving patients’ outcomes. However, overcoming certain challenges in the future is necessary to achieve widespread adoption and popularity of hand-held OCTA devices. These challenges include the lack of an in-built fixation target and the need for automated image segmentation and analysis programs, which will be further discussed in the following section (101).

### 5.2 Development of widefield OCTA

In the past, OCTA devices offered a limited field of view, often restricted to scan sizes of 3 × 3 mm or 6 × 6 mm. Hence, the ONH and macular angiograms had to be captured in separate scans. Information beyond these regions was often neglected. With the herald of widefield OCTA, imaging and analysis of larger retinal areas, including the peripapillary region and peripheral retina have been made possible. Recently, commercially available OCTA devices have been able to generate images with a field of view extending to 15 × 15 mm, simultaneously capturing both macula and ONH in a single acquisition (102). This is useful for detecting glaucoma in high myopic eyes, as high myopia affects both the central and peripheral retinas in widefield swept-source OCTA (103). In high myopic eyes with an elongated axial length, the retina is thinned and could mimic the structural thinning in glaucomatous eyes. This generates false positives, also known as “red disease” as seen in OCT deviation maps (104). Indeed, other myopic-related structural changes also hinder the accuracy of diagnosing glaucoma with OCT imaging including rotation, tilting, torsion, and peripapillary atrophy of the ONH, rendering the diagnosis of glaucoma challenge in high myopic eyes (105). To tackle this problem, widefield OCTA has been shown to offer better diagnostic ability for high myopia glaucoma. Hong et al. demonstrated that the specificity of widefield OCTA map was significantly higher than that of OCT widefield map (86.94% vs. 80.51%, *p* < 0.001) when used for diagnosing glaucoma in high myopic eyes, despite statistically similar sensitivity (87.28% vs. 87.49%, *p* = 0.078) (106). Hence, integrating a widefield OCTA map into clinical practice might help reduce false-positive findings in distinguishing between true glaucomatous structural impairment and myopia-related structural loss, which is often challenging for clinicians.

Regardless of its merits, widefield OCTA has its limitations. Capturing a wider area requires longer acquisition time, and thus, image acquisition is hindered by the patient’s blinking or inattention, leading to artifacts or low image quality. Further efforts are warranted to shorten acquisition time in the future.

### 5.3 Artificial intelligence and deep learning application in OCTA imaging

Imaging analysis programs based on artificial intelligence (AI) could enhance the accuracy and efficiency of OCTA imaging. These programs would allow instant quantification of vascular changes and identification of pathological features.

Using AI and deep learning (DL) in OCTA offers multiple applications and advantages. Learning algorithms can be trained to generate OCTA-like angiograms directly from a single structural OCT-B scan, compared to the need for multiple B scans in traditional means. This enables shorter OCTA scan acquisition times, thus reducing motion artifacts (107). Convolutional neuronal networks (CNN) can also be applied to reconstruct and improve OCTA image quality (107). Deep learning can further remove such artifacts as bulk motion artifacts from micro-saccades and shadow artifacts from vitreous floaters by comparing *en face* OCTA and OCT reflectance images to identify shadows (108). Moreover, deep learning-based AI quantification can replace the time-consuming manual calculation of OCTA features, such as VD and segmentation, non-perfusion areas, choroidal neovascularization, and retinal fluid. Eventually, computer-aided diagnostic (CAD) systems can facilitate disease diagnosis through combinations of biomarkers and other AI-generated parameters (107). Compared to traditional approaches such as logistic regression, CAD systems have demonstrated superior disease diagnosis and classification performance.

By utilizing large data sets and learning algorithms, AI-driven OCTA imaging enables more precise and timely detection of diseases, leading to personalized interventions for glaucoma patients with different subtypes and clinical severity. Integrating AI and deep learning into OCTA imaging is a revolutionary step that paves the way for comprehensive glaucoma management and ultimately better therapeutic outcomes. However, future endeavors are warranted to overcome the challenges in applying AI and deep learning in OCTA. For instance, AI-generated angiograms fail to capture some capillary details that can be retrieved from OCTA (109), and CAD diagnostic systems lack the ability to utilize features that have not been previously identified (107). To facilitate future advancements, it is crucial for researchers to acquire a comprehensive understanding of OCTA technology and data processing mechanisms, which is essential for designing learning algorithms that are capable of addressing existing limitations and enhancing the overall performance of OCTA.

## 6 Conclusion

OCTA has emerged as an invaluable non-invasive imaging modality in glaucoma diagnosis and management. OCTA offers insights into microvascular changes associated with glaucoma progression by providing detailed information on the retinal and optic nerve microvasculature. The clinical translation of OCTA opens up a promising avenue for early detection, disease progression monitoring, and treatment outcome assessment in glaucoma patients. Despite the limitations and challenges, ongoing research and technological advancements are expected to enhance the utility of OCTA in the clinical management of glaucoma. As we move forward, further studies are warranted to validate the clinical utility of OCTA, optimize imaging protocols, and possibly integrate this technology into our routine clinical practice to improve patient outcomes in the management of glaucoma.
